# Automated Generation of Personalized Shock Wave Lithotripsy Protocols: Treatment Planning Using Deep Learning

**DOI:** 10.2196/24721

**Published:** 2021-05-11

**Authors:** Zhipeng Chen, Daniel D Zeng, Ryan G N Seltzer, Blake D Hamilton

**Affiliations:** 1 Shenzhen Artificial Intelligence and Data Science Institute (Longhua) Longhua, Shenzhen China; 2 The State Key Laboratory of Management and Control for Complex Systems Institute of Automation Chinese Academy of Sciences Beijing China; 3 Translational Analytics and Statistics Tucson, AZ United States; 4 School of Medicine University of Utah Salt Lake City, UT United States

**Keywords:** nephrolithiasis, extracorporeal shock wave therapy, lithotripsy, treatment planning, deep learning, artificial intelligence

## Abstract

**Background:**

Though shock wave lithotripsy (SWL) has developed to be one of the most common treatment approaches for nephrolithiasis in recent decades, its treatment planning is often a trial-and-error process based on physicians’ subjective judgement. Physicians’ inexperience with this modality can lead to low-quality treatment and unnecessary risks to patients.

**Objective:**

To improve the quality and consistency of shock wave lithotripsy treatment, we aimed to develop a deep learning model for generating the next treatment step by previous steps and preoperative patient characteristics and to produce personalized SWL treatment plans in a step-by-step protocol based on the deep learning model.

**Methods:**

We developed a deep learning model to generate the optimal power level, shock rate, and number of shocks in the next step, given previous treatment steps encoded by long short-term memory neural networks and preoperative patient characteristics. We constructed a next-step data set (N=8583) from top practices of renal SWL treatments recorded in the International Stone Registry. Then, we trained the deep learning model and baseline models (linear regression, logistic regression, random forest, and support vector machine) with 90% of the samples and validated them with the remaining samples.

**Results:**

The deep learning models for generating the next treatment steps outperformed the baseline models (accuracy = 98.8%, F1 = 98.0% for power levels; accuracy = 98.1%, F1 = 96.0% for shock rates; root mean squared error = 207, mean absolute error = 121 for numbers of shocks). The hypothesis testing showed no significant difference between steps generated by our model and the top practices (*P*=.480 for power levels; *P*=.782 for shock rates; *P*=.727 for numbers of shocks).

**Conclusions:**

The high performance of our deep learning approach shows its treatment planning capability on par with top physicians. To the best of our knowledge, our framework is the first effort to implement automated planning of SWL treatment via deep learning. It is a promising technique in assisting treatment planning and physician training at low cost.

## Introduction

Shock wave lithotripsy (SWL, or extracorporeal shock wave lithotripsy) has been considered as a safe and effective noninvasive treatment option for nephrolithiasis since its introduction in early 1980s [1]. Reported SWL stone-free rates approach 74%-88% [2,3]; however, it is not without risk. Common contraindications to SWL include pregnancy, coagulopathy or use of platelet aggregation inhibitors, aortic aneurysms, severe untreated hypertension, and untreated urinary tract infections [4]. Failure of SWL treatment results in unnecessary exposure to various complications, such as loin pain, dysuria, analgesia, hematuria, and infection [3,5].

Given such risks, previous studies have identified proper patient selection, modifications in treatment technique, and employment of adjunctive measures as elements to improve SWL outcomes [6]. The treatment outcomes are strongly affected by a variety of preoperative patient characteristics (PPC), including BMI [7-9], stone location, overall stone burden [4], skin-to-stone distance [10,11], stone composition [12,13], stone density [14-17], and variation coefficients of stone density [18]. Various studies have also demonstrated that precise targeting [19,20] and tight coupling [21,22] increase fragmentation probability.

Appropriate control over shock wave delivery has a strong impact on treatment success and minimal complications. A treatment plan for shock wave delivery is a series of shock wave delivery steps with a specified power level, shock rate, and number of shocks; a successful sample SWL treatment plan is shown in Table 1. A plan precisely specifies step-by-step power levels, shock rates, and number of shocks. Each treatment step has a single power level, a constant shock rate, and shocks usually between 500-2500 [23-28]. Physicians are obliged to design plans that both deliver sufficient energy for breaking stones and minimize damage to body tissues. While the range of shock rates is typically 30-180 shocks/minute, a shock rate of 60-90 shocks/minute has been shown to improve efficacy [29-31] and decrease potential injury risks. The main reason is that the slower shock rate of 60-90 shocks/minute allows time for cavitation bubbles caused by the shock to disperse before the next shock arrives. Physicians can check stone fragmentation via x-ray. If the fragments of treated stones are ≤4 mm, they typically pass on their own without further treatment. An SWL treatment has to be stopped to reduce risks of tissue damage when the shock number reaches the maximum limit, even though the treated stone has not broken up.

**Table 1:** A sample shock wave lithotripsy (SWL) treatment plan.

Shock wave delivery steps	Power level	Shock rate (per minute)	Number of shocks
Step 1	1	120	100
Step 2	2	120	100
Step 3	3	120	100
Step 4	4	120	100
Step 5	5	120	100
Step 6	6	120	100
Step 7	7	120	100
Step 8	8	120	2300

Effective fragmentation leads to fewer shocks overall and therefore less damage to tissue [32,33]. In order to maximize treatment effect and control tissue damage, ramping protocols have been developed. The low-energy pretreatment allows for better pain management, thus preventing movement and subsequent decoupling of the shock head [34]. Clinical trials support that stepwise voltage ramping is associated with less tissue damage compared with a fixed maximal voltage protocol [23,25,26,35].

Although the strength, rates, and total number of shock waves are identified as the important factors of SWL treatment outcomes, there is no case-by-case guideline for physicians to optimize shock wave delivery protocols that take into account patient demographics and stone characteristics. The optimal energy delivery strategy remains controversial. In vitro and in vivo studies suggest that the strategy of ramping up shock wave energy is beneficial to improve fragmentation and stone clearance and limit renal damage, but clinical results are discordant [6,23]. In the current planning process, physicians adopt a trial-and-error approach to tune treatment plans. This approach involves nonintuitive iterations based on physicians’ subjective decisions. Inexperienced physicians using this method may be more apt to produce inefficient or ineffective treatment plans. Such dependence on physicians’ unique experience also leads to significant variability in the quality and consistency of treatment delivery. Moreover, different types of machines have different designs and different sources for generating shock waves. Therefore, an effective treatment plan for one machine may not transfer to a different machine.

As a result, SWL success rates are significantly different among physicians. Table 2 shows the percentiles of success rates of 171 physicians who recorded outcomes in the International Stone Registry, a database of accumulated treatment records for all patients treated within a national network of SWL services provided by Translational Analytics and Statistics, a lithotripsy service provider. Here, treatment success is defined as treated stone fragments ≤4 mm that typically pass on their own without further treatment. The top 20% of physicians have success rates higher than 94.3%, while the success rates of the bottom 20% of physicians are lower than 79.1%. Such variation indicates that the inexperience with and subjectivity of SWL treatment could lead to unnecessary damage to patients.

**Table 2:** Percentiles of treatment success rates.

Percentiles	Treatment success rates, %
Minimum	54.5
10th percentile	74.8
20th percentile	79.1
30th percentile	82.6
40th percentile	84.7
50th percentile	86.6
60th percentile	88.9
70th percentile	91.4
80th percentile	94.3
90th percentile	100
Maximum	100

Machine learning techniques have been applied in the planning process of high-quality personalized treatments, such as radiation therapies [36-39], chemotherapies [40,41] and diabetes treatments [42]. Most machine learning models only take independent vectors as inputs, so they are not suited to the sequential nature of SWL treatment plans. However, recurrent neural networks (RNNs) are naturally suited to temporal sequence inputs. Several variants like long short-term memory (LSTM) [43] and gated recurrent unit [44] have been developed for sequential features and applied to disease diagnosis [45,46]. Following these recent works, we aimed to validate the deep learning approach to generate next SWL treatment steps by learning the practices of top physicians and, based on the deep learning approach, develop a system to automatically produce personalized, unbiased, and consistent SWL treatment plans. The generated treatment plans can help physicians minimize the trial-and-error process and develop evidence-based personalized treatment based on PPC, including patient demographics and stone characteristics. An additional benefit is that this treatment planning framework can be generalized to different machine types, so physicians can easily adapt to new generations of SWL machines.

## Methods

### Data

To train and evaluate our models, we used a dataset of renal treatments with Storz SLX-T from the International Stone Registry provided by Translational Analytics and Statistics. Each treatment consisted of PPC and several treatment steps (ie, ternaries of a power level, a shock rate, and number of shocks). The power level ranged from 1 to 9. The options for shock rates were 60, 90, 120, and 180 shocks per minute. The maximum number of shocks was typically set at 3000 for renal stones. The PPC in our dataset included patient gender, age, stone location (one-hot encoding), stone size, mean arterial pressure before treatment, anticoagulant use, sedation use, whether multiple stones existed, and whether strapping was applied.

Our deep learning models were trained with the best treatment plans for obtaining the best planning capability. We selected 54 physicians in the top quartile of treatment success rates. These physicians had more than 91.4% treatment success rates. Then, we selected their successful treatment cases with no reported complications, in which they were stone free or had fragments ≤4 mm and typically passed on their own without further treatment. We identified 1216 cases in total and assumed these cases are the best practices in SWL treatment planning.

We then built the step dataset from the identified successful cases to train and evaluate the step generation model. We identified steps by power level change or shock rate change and limited the number of shocks to 1000 for each step, a natural step length in previous literature [25]. If more than 1000 shocks were delivered under the same power level and the same shock rate, we broke them into multiple steps with 1000 shocks maximum.

Then, we exhaustively decomposed each case into samples by step for the step generation task, where the ternary of each step was generated by its previous steps and PPC. An *n*-step treatment case was decomposed into *n* – 1 samples: we used the first *i* step(s) and PPC as the model inputs and the power level, shock rate, and number of shocks in the (*i* + 1)th step as the model outputs, where 0 < *i* < *n*. For example, the SWL treatment case in Table 1 that consisted of 10 steps after the last 2300 shocks at power level 8 was split into 3 steps: (1) power level = 8, shock rate = 120, number of shocks = 1000; (2) power level = 8, shock rate = 120, number of shocks = 1000; and (3) power level = 8, shock rate = 120, number of shocks = 300. Then, we decomposed this case into 9 samples: (1) The input is the first step (power level = 1, shock rate = 120, number of shocks = 100) and PPC, and the output is the second step (power level = 2, shock rate = 120, number of shocks = 100); (2) the input is the first 2 steps and PPC, and the output is the third step (power level = 3, shock rate = 120, number of shocks = 100); …; and (9) the input is the first 9 steps and PPC, and the output is the last step (power level = 8, shock rate = 120, number of shocks = 300).

At last, we constructed 8583 samples for step generation. We randomly chose 90% of the samples for model training and used the remaining samples for validation. In the data split, we enforced that samples from the same treatment case were only contained within the same split.

### Deep Learning for Step Generation

**Figure 1:**
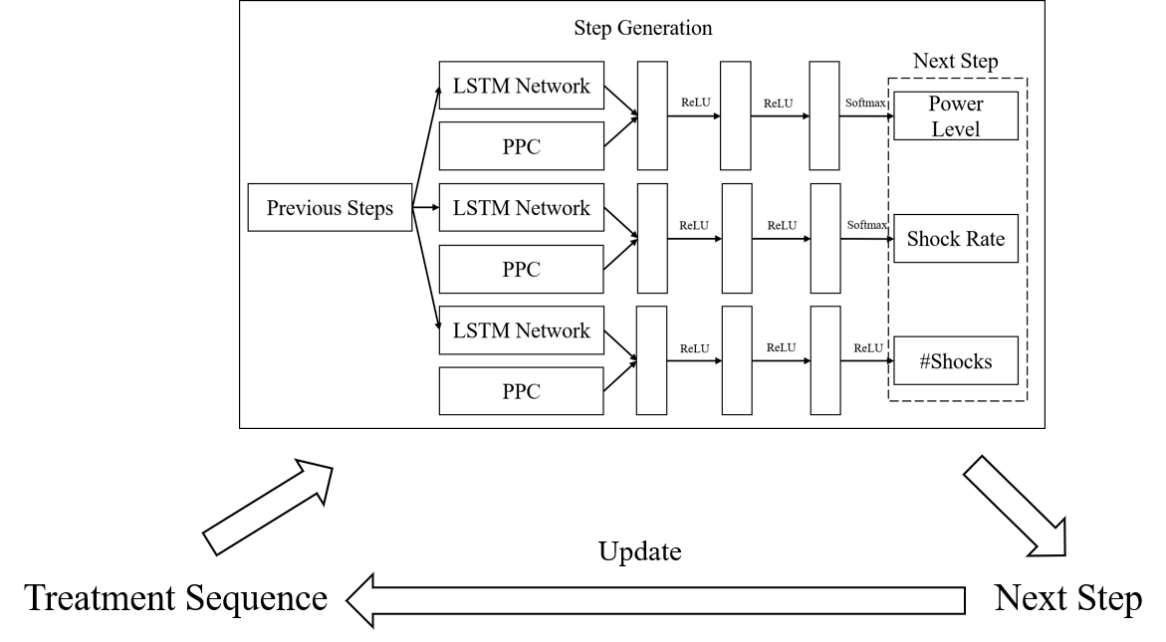
The framework for automated shock wave lithotripsy (SWL) treatment planning. LSTM: long short-term memory; PPC: preoperative patient characteristics; ReLU: rectifier linear unit.

We first built deep neural networks to separately generate power levels, shock rates, and numbers of shocks for the next steps, given previous steps and PPC (Figure 1). Most off-the-shelf machine learning models only take inputs represented as independent vectors rather than a sequence of previous steps. However, RNNs are naturally suited to temporal sequence inputs, so we adopted an RNN variant, the LSTM model [43], which can keep track of arbitrary long-term dependencies in the input sequences, to encode the treatment sequences to vectors. More specifically, assume the *i*-th step is encoded as a vector *x_i_*, then the LSTM model is defined iteratively as follows:



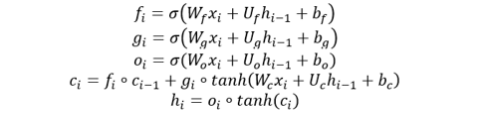



where the initial values *c_0_* and *h_0_* are zero vectors, ° denotes the element-wise product, σ is the sigmoid function, and *h_1_* is the representation of the first *i* treatment steps.

Then, the encoded previous steps were concatenated to PPC vectors and fed to deep neural networks. In our implementation, we used 2 fully connected layers with a rectifier linear unit (ReLU) function as activation functions, because ReLU functions are nonsaturated and make the model less likely to overfit [47]. At last, we used different classifiers or regressors to generate power levels, shock rates and shock numbers. The formula are as follows.



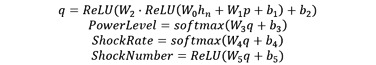



where *h_n_* is the *n* previous steps encoded by LSTM, and *p* denotes the PPC vector. The classifiers at the end of the networks were softmax functions for generating power level and shock rate because they are categorical, and we used categorical cross-entropy as the loss functions; for shock number generation in which the output is an integer, we used ReLU as the regressor and mean squared error (MSE) as the loss function. For all the deep neural networks, we chose the Adam SGD optimizer [48,49] in model training.

### Statistical Analysis

We hypothesized that the deep learning approach is comparable to the treatment practices of top physicians and that it outperforms machine learning models which do not take treatment sequences as inputs. Thus, we compared the performance of the deep learning model and other up-to-date machine learning models.

Three classical machine learning approaches were selected as baselines for generating power level, shock rate, and number of shocks, respectively. We used logistic regression, random forest classifier (RFC), and support vector classifier (SVC) as the baseline models for power level generation and shock rate generation. We chose linear regression, random forest regression (RFR), and support vector regression (SVR) as the baseline models to generate the number of shocks. As these baseline models could not be fed with sequential data directly, the features for the baseline models were (1) the average power level, average shock rate, and average number of shocks in previous steps; (2) the power level, shock rate, and number of shocks in the last step; and (3) PPC.

We trained the deep learning models and baseline models with 90% of the samples. Then, we validated them with the remaining samples and calculated evaluation metrics. In the multiclass tasks of power level generation and shock rate generation, we used accuracy, macro-averaged precision, macro-averaged recall, and macro-averaged F1 score as the evaluation metrics [50,51]. Accuracy was defined as a ratio of correctly generated observations to the total observations. Suppose the number of categories is *n* and the confusion matrix of a classifier is a *n* x *n* matrix *C*, where *C_i j_* is the number of samples that is labeled as *i* but generated as *j*, then the accuracy is defined as



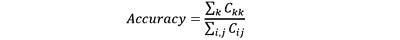



The precision and recall of category *k* are defined as



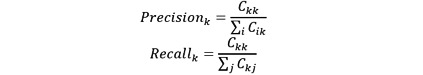



Macro-averaged precision and recall are the average of precisions and recalls for all categories:



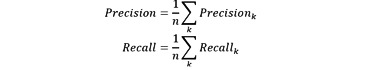



The F1 score of category *k* is defined as the harmonic mean of precision and recall of category *k*



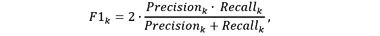



and macro-averaged F1 score is defined as the average of F1 scores for all categories:



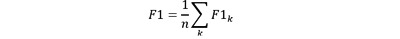



Because the number of shocks is an integer, we used the root mean squared error (RMSE) and mean absolute error (MAE) as the metrics to evaluate the models generating the number of shocks and to measure the average magnitude of errors. At last, we conducted paired *t* test to detect the difference between treatment steps generated by machine learning models and treatment practices of top physicians.

## Results

The deep learning models generated high-quality treatment steps and outperformed the baselines, as summarized in Tables 3-5. In power level generation (Table 3), the accuracy of the deep learning model was 0.988, and the precision, recall, and F1 scores were all 0.980. The best baseline was the SVC, for which the accuracy was 0.981, precision was 0.969, recall was 0.976, and F1 score was 0.972, lower than the performance of the deep learning model. For shock rate generation (Table 4), our model achieved an F1 score of 0.960 along with an accuracy of 0.981, precision of 0.963, and recall of 0.957. Among the baseline models, the logistic regression performed the best in accuracy and precision, at 0.978 and 0.932, respectively, while the RFC had the best recall and F1 score, at 0.986 and 0.956, respectively. Though the recall of the RFC and the logistic regression was better than that of the deep learning model, the accuracy, precision, and F1 score of our proposed model outperformed all the baseline models. The RMSE of the generation of the number of shocks (Table 5) by the deep learning model was 207, about 19% less than the best baseline model RFR. The MAE of the deep learning model was 121, about 23% less than the best baseline model.

**Table 3:** Model performance in power level generation.

Model	Accuracy	Precision	Recall	F1	*t* statistic	*P* value
Deep learning	0.988	0.980	0.980	0.980	0.707	.480
Logistic regression	0.974	0.964	0.964	0.964	1.257	.209
RFC^a^	0.708	0.823	0.859	0.803	4.976	<.001
SVC^b^	0.981	0.969	0.976	0.972	2.205	.028

^a^RFC: random forest classifier.

^b^SVC: support vector classifier.

**Table 4:** Model performance in shock rate generation.

Model	Accuracy	Precision	Recall	F1	*t* statistic	*P* value
Deep learning	0.981	0.963	0.957	0.960	0.277	.782
Logistic regression	0.978	0.932	0.960	0.945	2.331	.020
RFC^a^	0.952	0.930	0.986	0.956	2.064	.039
SVC^b^	0.976	0.926	0.956	0.939	2.510	.012

^a^RFC: random forest classifier.

^b^SVC: support vector classifier.

**Table 5:** Model performance in shock number generation.

Model	RMSE^a^	MAE^b^	*t* statistic	*P* value
Deep learning	207	121	0.350	.727
Linear regression	265	206	0.917	.359
RFR^c^	255	158	0.628	.530
SVR^d^	350	173	9.427	<.001

^a^RMSE: root mean squared error.

^b^MAE: mean absolute error.

^c^RFR: random forest regression.

^d^SVC: support vector regression.

The analysis also tested the difference between the generated step and the ground truth. In the paired *t* test result, there was no evidence indicating a difference between the generated steps of the deep learning model and treatment steps planned by top physicians, while the outputs of some baseline models significantly deviated from the ground truth. The power levels generated by the RFC and SVC, the shock rates generated by all the baseline models, and the numbers of shocks generated by the SVR were significantly different from the treatment steps in the successful SWL cases of top physicians.

Furthermore, we analyzed the performance of the deep learning models on samples of various treatment sequence lengths to gain a better understanding of how the treatment sequence information could aid decision making. We partitioned the validation dataset into 9 sets by the number of previous treatment steps and summarized the validation results in Tables 6-8. As shown in Table 6, the deep learning model was able to perfectly generate power levels when previous treatment steps were fewer than 6. As the number of previous treatment steps increases, the treatment becomes more complicated and leads to lower performance of power level generation by the deep learning model. The deep learning model reached the lowest accuracy (accuracy = 0.875) and lowest recall (recall = 0.500) in samples containing 9 previous treatment steps and the lowest precision (precision = 0.873) and lowest F1 score (F1 = 0.888) in samples containing 8 previous treatment steps. Similarly, the deep learning model generated highly accurate shock rates in samples with previous treatment steps fewer than 6; the model reached the lowest accuracy (accuracy = 0.889), lowest precision (precision = 0.857), and lowest recall (recall = 0.631) in samples containing 7 previous treatment steps and the lowest F1 score (F1 = 0.861) in samples containing 6 previous treatment steps (Table 7). For the performance of generating the number of shocks (Table 8), the RMSE and MAE generally increased as the number of previous treatment steps increased, and the maximum number of errors appeared in samples with 5 previous treatment steps (RMSE = 365; MAE = 310). The results show the excellent performance of deep learning models in step generation in the first 4-6 steps, where most successful cases end. It reflects the reliability of deep learning models in aiding treatment decision making. Longer treatment lengths typically indicate treatment difficulties; even our deep learning models cannot generate treatment steps with high accuracy in these rare cases.

**Table 6:** Power level generation performance in samples containing different numbers of previous treatment steps.

Number of previous treatment steps	Accuracy	Precision	Recall	F1
1	1.000	1.000	1.000	1.000
2	1.000	1.000	1.000	1.000
3	1.000	1.000	1.000	1.000
4	1.000	1.000	1.000	1.000
5	1.000	1.000	1.000	1.000
6	0.983	0.980	0.980	0.980
7	0.926	0.915	0.939	0.925
8	0.889	0.873	0.914	0.888
9	0.875	0.875	0.500	0.933

**Table 7:** Shock rate generation performance in samples containing different numbers of previous treatment steps.

Number of previous treatment steps	Accuracy	Precision	Recall	F1
1	1.000	1.000	1.000	1.000
2	0.992	0.997	0.972	0.984
3	1.000	1.000	1.000	1.000
4	1.000	1.000	1.000	1.000
5	0.992	0.997	0.974	0.985
6	0.975	0.976	0.802	0.861
7	0.889	0.857	0.631	0.888
8	0.917	0.864	0.642	0.902
9	1.000	1.000	1.000	1.000

**Table 8:** Performance of the generation of the number of shocks in samples containing different numbers of previous treatment steps.

Number of previous treatment steps	RMSE^a^	MAE^b^
1	31	25
2	32	17
3	34	24
4	139	60
5	365	310
6	317	233
7	273	190
8	275	242
9	99	76

^a^RMSE: root mean squared error.

^b^MAE: mean absolute error.

The validation showed that the capability of the deep learning model for step generation is on par with that of top physicians. Based on the high-quality step generation, we generated treatment plans by iteratively generating steps with the trained models (Figure 1). We started from an empty treatment sequence. We fed PPC and the current treatment sequence into the step generation model. The generated next step was then added to the current treatment sequence. We repeated such a process until the total number of shocks reached the upper limit. If a physician confirms stone fragmentation via x-ray before reaching the maximum limit of the number of shocks, they can stop immediately; if the number of shocks reaches the maximum limit, the physician has to stop for risk control. Thus, the generated treatment sequence is enough to guide practice. The specifications of individual lithotripters limit the maximum number of shocks per session to 2000-4500 [4], and for the majority of treatments of upper ureteral and renal stones, the range is 2000-3500 [52]. We used 3000 as the upper limit in our implementation, which is a typical shock limit in renal stone treatment practices and can be adjusted according to shock wave generating machines.

## Discussion

### Principal Findings

Previous literature has shown a series of work on standardizing SWL treatment [2,53]; however, energy delivery is still controversial and unclear [4,6], relying on physicians’ subjective judgement. Manual treatment design is significantly affected by nonstandardizable radiographic appearance of stones, bias to a low power level for fear of complications, and preconceived expectations. Our study utilized deep learning to generate treatment steps and developed a framework for automated SWL treatment planning.

The analysis results revealed that deep learning models for treatment step generation effectively learn from SWL treatment plans and achieve the step generation capability of top physicians. The performance comparison indicated that utilization of a previous treatment sequence in deep learning improves the quality of generated steps. By iteratively generating treatment steps, our automated planning framework can avoid human biases and generate personalized, high-quality, and consistent SWL treatment plans based on PPC, including patient demographics and stone characteristics. With the help of these automatically generated treatment plans, physicians can minimize the trial-and-error process and implement evidence-based personalized treatment. This framework can be generalized to different machine types, so physicians can easily adapt to new generations of SWL machines.

### Limitations

Our proposed model only learns and imitates the best practices, but cannot perform better than them. Even the best physician cannot plan successful SWL treatment plans for all cases, so successful difficult cases, including those requiring long treatment sequences, are rare for model training. Therefore, our model may be good at planning easier cases, but less adept in rare difficult cases, similar to physicians’ actual practice. As the treatment cases, especially successful difficult cases, accumulate, our model is likely to gain an expert-level planning capability to handle difficult cases.

Due to data limitations, we were only able to consider a small set of patient demographics and stone characteristics. However, our framework can be easily extended to utilize a larger set of parameters than has previously been used. Moreover, the data are retrospective. Therefore, clinical studies are warranted to confirm the effectiveness and efficiency of this framework.

### Conclusions

To the best of our knowledge, our framework is the first effort to implement automated planning of SWL treatment via deep learning. Its assistance for inexperienced urologists in designing SWL treatment plans is useful in both SWL treatment planning and physician training. While the applications of machine learning in diagnosis are becoming more mature, few studies exist in automated treatment plan generation. Our approach is a step forward in exerting the potential of machine learning in medical sciences.

## Abbreviations

LSTM: long short-term memory
MAE: mean absolute error
MSE: mean squared error
PPC: preoperative patient characteristics
ReLU: rectifier linear unit
RFC: random forest classifier
RFR: random forest regression
RMSE: root mean squared error
RNN: recurrent neural network
SVC: support vector classifier
SVR: support vector regression
SWL: shock wave lithotripsy

## References

[1] Chaussy C, Brendel W, Schmiedt E (1980). Extracorporeally induced destruction of kidney stones by shock waves. Lancet.

[2] Assimos D, Krambeck A, Miller NL, Monga M, Murad MH, Nelson CP, Pace KT, Pais VM, Pearle MS, Preminger GM, Razvi H, Shah O, Matlaga BR (2016). Surgical Management of Stones: American Urological Association/Endourological Society Guideline, PART I. J Urol.

[3] Al-Marhoon MS, Shareef O, Al-Habsi IS, Al Balushi AS, Mathew J, Venkiteswaran KP (2013). Extracorporeal Shock-wave Lithotripsy Success Rate and Complications: Initial Experience at Sultan Qaboos University Hospital. Oman Med J.

[4] Reynolds LF, Kroczak T, Pace KT (2018). Indications and contraindications for shock wave lithotripsy and how to improve outcomes. Asian J Urol.

[5] Skolarikos A, Alivizatos G, de la Rosette J (2006). Extracorporeal shock wave lithotripsy 25 years later: complications and their prevention. Eur Urol.

[6] McClain PD, Lange JN, Assimos DG (2013). Optimizing shock wave lithotripsy: a comprehensive review. Rev Urol.

[7] Pareek G, Armenakas NA, Panagopoulos G, Bruno JJ, Fracchia JA (2005). Extracorporeal shock wave lithotripsy success based on body mass index and Hounsfield units. Urology.

[8] Perks AE, Schuler TD, Lee J, Ghiculete D, Chung D, D'A Honey RJ, Pace KT (2008). Stone attenuation and skin-to-stone distance on computed tomography predicts for stone fragmentation by shock wave lithotripsy. Urology.

[9] Hatiboglu G, Popeneciu V, Kurosch M, Huber J, Pahernik S, Pfitzenmaier J, Haferkamp A, Hohenfellner M (2011). Prognostic variables for shockwave lithotripsy (SWL) treatment success: no impact of body mass index (BMI) using a third generation lithotripter. BJU Int.

[10] Wiesenthal JD, Ghiculete D, Ray AA, Honey RJD, Pace KT (2011). A clinical nomogram to predict the successful shock wave lithotripsy of renal and ureteral calculi. J Urol.

[11] Patel T, Kozakowski K, Hruby G, Gupta M (2009). Skin to stone distance is an independent predictor of stone-free status following shockwave lithotripsy. J Endourol.

[12] Dretler SP (1994). Special article: calculus breakability--fragility and durility. J Endourol.

[13] Ringdén Ida, Tiselius H (2007). Composition and clinically determined hardness of urinary tract stones. Scand J Urol Nephrol.

[14] Ouzaid I, Al-qahtani S, Dominique S, Hupertan V, Fernandez P, Hermieu J, Delmas V, Ravery V (2012). A 970 Hounsfield units (HU) threshold of kidney stone density on non-contrast computed tomography (NCCT) improves patients' selection for extracorporeal shockwave lithotripsy (ESWL): evidence from a prospective study. BJU Int.

[15] El-Nahas AR, El-Assmy AM, Mansour O, Sheir KZ (2007). A prospective multivariate analysis of factors predicting stone disintegration by extracorporeal shock wave lithotripsy: the value of high-resolution noncontrast computed tomography. Eur Urol.

[16] Joseph P, Mandal A, Singh S, Mandal P, Sankhwar S, Sharma S (2002). Computerized Tomography Attenuation Value of Renal Calculus: Can It Predict Successful Fragmentation of the Calculus by Extracorporeal Shock Wave Lithotripsy? A Preliminary Study. Journal of Urology.

[17] Abdelhamid M, Mosharafa AA, Ibrahim H, Selim HM, Hamed M, Elghoneimy MN, Salem HK, Abdelazim MS, Badawy H (2016). A Prospective Evaluation of High-Resolution CT Parameters in Predicting Extracorporeal Shockwave Lithotripsy Success for Upper Urinary Tract Calculi. J Endourol.

[18] Yamashita S, Kohjimoto Y, Iguchi T, Nishizawa S, Iba A, Kikkawa K, Hara I (2017). Variation Coefficient of Stone Density: A Novel Predictor of the Outcome of Extracorporeal Shockwave Lithotripsy. J Endourol.

[19] Bohris C, Stief CG, Strittmatter F (2016). Improvement of SWL Efficacy: Reduction of the Respiration-Induced Kidney Motion by Using an Abdominal Compression Plate. J Endourol.

[20] Honey RJ, Healy M, Yeung M, Psihramis KE, Jewett MA (1992). The Use of an Abdominal Compression Belt to Reduce Stone Movement During Extracorporeal Shock Wave Lithotripsy. Journal of Urology.

[21] Pishchalnikov YA, Neucks JS, VonDerHaar RJ, Pishchalnikova IV, Williams JC, McAteer JA (2006). Air pockets trapped during routine coupling in dry head lithotripsy can significantly decrease the delivery of shock wave energy. J Urol.

[22] Jain A, Shah TK (2007). Effect of air bubbles in the coupling medium on efficacy of extracorporeal shock wave lithotripsy. Eur Urol.

[23] Rabah DM, Mabrouki MS, Farhat KH, Seida MA, Arafa MA, Talic RF (2017). Comparison of escalating, constant, and reduction energy output in ESWL for renal stones: multi-arm prospective randomized study. Urolithiasis.

[24] Connors BA, Evan AP, Handa RK, Blomgren PM, Johnson CD, Liu Z, Lingeman JE (2016). Using 300 Pretreatment Shock Waves in a Voltage Ramping Protocol Can Significantly Reduce Tissue Injury During Extracorporeal Shock Wave Lithotripsy. J Endourol.

[25] Lambert EH, Walsh R, Moreno MW, Gupta M (2010). Effect of escalating versus fixed voltage treatment on stone comminution and renal injury during extracorporeal shock wave lithotripsy: a prospective randomized trial. J Urol.

[26] Handa RK, McAteer JA, Connors BA, Liu Z, Lingeman JE, Evan AP (2012). Optimising an escalating shockwave amplitude treatment strategy to protect the kidney from injury during shockwave lithotripsy. BJU Int.

[27] McAteer JA, Evan AP, Williams JC, Lingeman JE (2009). Treatment protocols to reduce renal injury during shock wave lithotripsy. Curr Opin Urol.

[28] Honey RJD, Ray AA, Ghiculete D, Pace KT, University of Toronto Lithotripsy Associates (2010). Shock wave lithotripsy: a randomized, double-blind trial to compare immediate versus delayed voltage escalation. Urology.

[29] Pishchalnikov YA, McAteer JA, Williams JC, Pishchalnikova IV, Vonderhaar RJ (2006). Why stones break better at slow shockwave rates than at fast rates: in vitro study with a research electrohydraulic lithotripter. J Endourol.

[30] Pishchalnikov YA, McAteer JA, Williams JC (2008). Effect of firing rate on the performance of shock wave lithotriptors. BJU Int.

[31] Kang DH, Cho KS, Ham WS, Lee H, Kwon JK, Choi YD, Lee JY (2016). Comparison of High, Intermediate, and Low Frequency Shock Wave Lithotripsy for Urinary Tract Stone Disease: Systematic Review and Network Meta-Analysis. PLoS One.

[32] Delius M, Jordan M, Eizenhoefer H, Marlinghaus E, Heine G, Liebich HG, Brendel W (1988). Biological effects of shock waves: Kidney haemorrhage by shock waves in dogs—Administration rate dependence. Ultrasound in Medicine & Biology.

[33] Willis LR, Evan AP, Connors BA, Shao Y, Blomgren PM, Pratt JH, Fineberg NS, Lingeman JE (2005). Shockwave lithotripsy: dose-related effects on renal structure, hemodynamics, and tubular function. J Endourol.

[34] Mobley TB, Myers DA, Grine WB, Jenkins JM, Jordan WR (1993). Low Energy Lithotripsy with the Lithostar: Treatment Results with 19,962 Renal and Ureteral Calculi. Journal of Urology.

[35] Skuginna V, Nguyen DP, Seiler R, Kiss B, Thalmann GN, Roth B (2016). Does Stepwise Voltage Ramping Protect the Kidney from Injury During Extracorporeal Shockwave Lithotripsy? Results of a Prospective Randomized Trial. Eur Urol.

[36] Fan J, Wang J, Chen Z, Hu C, Zhang Z, Hu W (2019). Automatic treatment planning based on three-dimensional dose distribution predicted from deep learning technique. Med Phys.

[37] Smith WP, Kim M, Holdsworth C, Liao J, Phillips MH (2016). Personalized treatment planning with a model of radiation therapy outcomes for use in multiobjective optimization of IMRT plans for prostate cancer. Radiat Oncol.

[38] Nicolae A, Morton G, Chung H, Loblaw A, Jain S, Mitchell D, Lu L, Helou J, Al-Hanaqta M, Heath E, Ravi A (2017). Evaluation of a Machine-Learning Algorithm for Treatment Planning in Prostate Low-Dose-Rate Brachytherapy. Int J Radiat Oncol Biol Phys.

[39] Mak RH, Endres MG, Paik JH, Sergeev RA, Aerts H, Williams CL, Lakhani KR, Guinan EC (2019). Use of Crowd Innovation to Develop an Artificial Intelligence-Based Solution for Radiation Therapy Targeting. JAMA Oncol.

[40] Lee S, Celik S, Logsdon BA, Lundberg SM, Martins TJ, Oehler VG, Estey EH, Miller CP, Chien S, Dai J, Saxena A, Blau CA, Becker PS (2018). A machine learning approach to integrate big data for precision medicine in acute myeloid leukemia. Nat Commun.

[41] Lin H, Wei N, Chou T, Lin C, Lan Y, Chang S, Wang H, Yang S, Chen W, Lin T, Lin J, Jiang J (2017). Building personalized treatment plans for early-stage colorectal cancer patients. Oncotarget.

[42] Doubleday K, Zhou H, Fu H, Zhou J (2018). An Algorithm for Generating Individualized Treatment Decision Trees and Random Forests. J Comput Graph Stat.

[43] Hochreiter S, Schmidhuber J (1997). Long short-term memory. Neural Comput.

[44] Cho K, van Merrienboer B, Gulcehre C, Bahdanau D, Bougares F, Schwenk H, Bengio Y (2014). Learning phrase representations using RNN encoder-decoder for statistical machine translation. Cornell University.

[45] Gulshan V, Peng L, Coram M, Stumpe MC, Wu D, Narayanaswamy A, Venugopalan S, Widner K, Madams T, Cuadros J, Kim R, Raman R, Nelson PC, Mega JL, Webster DR (2016). Development and Validation of a Deep Learning Algorithm for Detection of Diabetic Retinopathy in Retinal Fundus Photographs. JAMA.

[46] Choi E, Schuetz A, Stewart WF, Sun J (2017). Using recurrent neural network models for early detection of heart failure onset. J Am Med Inform Assoc.

[47] Xu L, Choy CS, Li YW (2016). Deep sparse rectifier neural networks for speech denoising.

[48] Kingma DP, Ba J (2014). Adam: A method for stochastic optimization. Cornell University.

[49] Reddi SJ, Kale S, Kumar S (2019). On the convergence of Adam and beyond. Cornell University.

[50] Manning CD, Raghavan P, Schütze H (2010). Natural language engineering. Introduction to information retrieval.

[51] Narasimhan H, Pan W, Kar P, Protopapas P, Ramaswamy HG (2017). Optimizing the multiclass F-measure via biconcave programming.

[52] Rassweiler JJ, Knoll T, Köhrmann KU, McAteer JA, Lingeman JE, Cleveland RO, Bailey MR, Chaussy C (2011). Shock wave technology and application: an update. Eur Urol.

[53] Türk C, Petřík A, Sarica K, Seitz C, Skolarikos A, Straub M, Knoll T (2016). EAU Guidelines on Interventional Treatment for Urolithiasis. Eur Urol.

